# Peptides and Food Intake

**DOI:** 10.3389/fendo.2014.00058

**Published:** 2014-04-24

**Authors:** Carmen Sobrino Crespo, Aránzazu Perianes Cachero, Lilian Puebla Jiménez, Vicente Barrios, Eduardo Arilla Ferreiro

**Affiliations:** ^1^Biochemistry and Molecular Biology Unit, Department of Systems Biology, Faculty of Medicine, University of Alcalá, Alcalá de Henares, Spain; ^2^Department of Endocrinology, Hospital Infantil Universitario Niño Jesús, Instituto de Investigación La Princesa, Madrid, Spain; ^3^Centro de Investigación Biomédica en Red Fisiopatología Obesidad y Nutrición, Instituto de Salud Carlos III, Madrid, Spain

**Keywords:** peptides, orexigenic, ghrelin, anorexigenic, insulin

## Abstract

The mechanisms for controlling food intake involve mainly an interplay between gut, brain, and adipose tissue (AT), among the major organs. Parasympathetic, sympathetic, and other systems are required for communication between the brain satiety center, gut, and AT. These neuronal circuits include a variety of peptides and hormones, being ghrelin the only orexigenic molecule known, whereas the plethora of other factors are inhibitors of appetite, suggesting its physiological relevance in the regulation of food intake and energy homeostasis. Nutrients generated by food digestion have been proposed to activate G-protein-coupled receptors on the luminal side of enteroendocrine cells, e.g., the L-cells. This stimulates the release of gut hormones into the circulation such as glucagon-like peptide-1 (GLP-1), oxyntomodulin, pancreatic polypeptides, peptide tyrosine tyrosine, and cholecystokinin, which inhibit appetite. Ghrelin is a peptide secreted from the stomach and, in contrast to other gut hormones, plasma levels decrease after a meal and potently stimulate food intake. Other circulating factors such as insulin and leptin relay information regarding long-term energy stores. Both hormones circulate at proportional levels to body fat content, enter the CNS proportionally to their plasma levels, and reduce food intake. Circulating hormones can influence the activity of the arcuate nucleus (ARC) neurons of the hypothalamus, after passing across the median eminence. Circulating factors such as gut hormones may also influence the nucleus of the tractus solitarius (NTS) through the adjacent circumventricular organ. On the other hand, gastrointestinal vagal afferents converge in the NTS of the brainstem. Neural projections from the NTS, in turn, carry signals to the hypothalamus. The ARC acts as an integrative center, with two major subpopulations of neurons influencing appetite, one of them coexpressing neuropeptide Y and agouti-related protein (AgRP) that increases food intake, whereas the other subpopulation coexpresses pro-opiomelanocortin (POMC) and cocaine and amphetamine-regulated transcript that inhibits food intake. AgRP antagonizes the effects of the POMC product, α-melanocyte-stimulating hormone (α-MSH). Both populations project to areas important in the regulation of food intake, including the hypothalamic paraventricular nucleus, which also receives important inputs from other hypothalamic nuclei.

## Introduction

Nutrients created by the digestion of food are proposed to activate G-protein-coupled receptors on the luminal side of enteroendocrine cells, e.g., the L-cells. This stimulates the release of gut hormones. The hormones released from the gut and the adipose tissue (AT) play an important role in the regulation of food intake and energy expenditure (1).

Many circulating signals, including gut hormones, can influence the activity of the arcuate nucleus (ARC) neurons directly, after passing across the median eminence. The ARC is adjacent to the median eminence, a circumventricular organ with fenestrated capillaries and hence an incomplete blood–brain barrier (BBB) (2). The ARC plays a crucial role in the regulation of food intake and energy homeostasis. The ARC contains two populations of neurons with opposing effects on food intake (3). Medially located orexigenic neurons express neuropeptide Y (NPY) and agouti-related protein (AgRP) (4, 5). Anorexigenic neurons (i.e., those inhibiting appetite) in the lateral ARC express alpha-melanocyte-stimulating hormone (α-MSH) derived from pro-opiomelanocortin (POMC) and cocaine and amphetamine-regulated transcript (CART) (6). The balance between the activities of these neuronal circuits is critical to body weight regulation.

In contrast, other peripheral signals influence the hypothalamus indirectly via afferent neuronal pathways and brainstem circuits. In this context, the gastrointestinal vagal afferents are activated by mechanoreceptors and chemoreceptors, and converge in the nucleus of the tractus solitaries (NTS) of the brainstem. Neuronal projections from the NTS, in turn, carry signals to the hypothalamus (1, 7). Gut hormones also alter the activity of the ascending vagal pathway from the gut to the brainstem. In the case of ghrelin and peptide tyrosine tyrosine (PYY), there is some evidence for a direct action of both on the ARC and an action via the vagus nerve and brainstem.

The axons of these neurons project to “second-order” neurons, located in part in the paraventricular nucleus (PVN), where the anorexigenic substances thyrotropin-releasing hormone (TRH), corticotropin-releasing hormone (CRH), and oxytocin are secreted, and in part, in the lateral hypothalamic (LH) and perifornical area (PFA), where the orexant molecules melanin-concentrating hormone (MCH) and orexins are produced. When adipose signals reach the ARC, anorexigenic peptides are released which activate a catabolic circuit. In contrast, the activation of the anabolic pathway leads to the release of orexigenic peptides and occurs when adiposity signal concentrations in the brain are low, thus indicating the urgency to replenish fuel stores (8).

## Peripheral Gut Hormones

### Orexigenic peptides

#### Ghrelin

Ghrelin is a peptide consisting of 28 amino acids and is unusual among peptide hormones in that Ser3 is *n*-octanoylated.

Ghrelin is present in X/A-like cells, which account for approximately 20% of the endocrine cell population in adult oxyntic glands (9). Ghrelin-immunoreactive cells are also found in the duodenum, jejunum, ileum, and colon. In the intestine, the ghrelin concentration gradually decreases from the duodenum to the colon. This peptide is also secreted from other organs such as the hypothalamus and pancreas of rats and, in addition, ghrelin mRNA is expressed in various organs (10, 11). The best known factor for the regulation of ghrelin secretion is feeding. The plasma ghrelin concentration increases when fasting, and decreases after food intake. The factors involved in the regulation of ghrelin secretion have not yet been identified. Blood glucose levels may be a most probable candidate; thus, oral or intravenous administration of glucose decreasing plasma ghrelin concentrations exhibits a nocturnal increase and are low in obese people and high in lean people (12–18). Exogenous growth hormone (GH) decreases stomach ghrelin mRNA expression and plasma ghrelin concentration, but does not affect stomach ghrelin stores (19).

The localization of ghrelin receptors on vagal afferent neurons in the rat nodose ganglion suggests that ghrelin signals from the stomach are transmitted to the brain via the vagus nerve (20, 21). In summary, ghrelin is secreted primarily from the stomach in response to hunger and starvation, circulates in the blood and serves as a peripheral signal, informing the central nervous system (via vagus nerve) to stimulate feeding. Ghrelin-containing neurons are also found in the ARC of the hypothalamus, a region involved in appetite regulation (1). In fact, intracerebroventricular (ICV) injection of ghrelin increases cumulative food intake and decreases energy expenditure, resulting in body weight gain (21–24). To stimulate the release of the orexigenic peptides, ghrelin-containing neurons send efferent fibers onto NPY and AgRP-expressing neurons. On the other hand, to suppress the release of the anorexigenic peptide, ghrelin-containing neurons send efferent fibers onto POMC neurons. The ARC is also a target of leptin, an appetite-suppressing hormone produced in AT (25). Leptin directly inhibits the appetite-stimulating effects of NPY and AgRP, whereas hypothalamic ghrelin blocks leptin-induced reduction of feeding. Thus, ghrelin and leptin have a competitive interaction in the regulation of feeding.

Ghrelin is only a hunger signal from peripheral tissues. Intravenous and sub-cutaneous injections of ghrelin increase food intake; likewise, peripherally injected ghrelin stimulates hypothalamic neurons and food intake (22, 23, 26–28). Because the rate at which peripheral ghrelin passes the BBB has been shown to be very low, peripheral ghrelin must activate the appropriate hypothalamic regions via an indirect pathway (29).

In addition, it has been reported that the enzyme ghrelin *O*-acyl transferase, essential for ghrelin acylation, is modulated by nutrient availability, depends on specific dietary lipids as acylation substrates. Thus, this mechanism may act as a nutrient sensor by using absorbable fatty acids to signal to the brain that high caloric food is available, leading to optimization of nutrient partitioning (30).

### Anorexigenic agents

#### Peptide tyrosine tyrosine

Peptide tyrosine tyrosine has 36 amino acids, contains several tyrosine residues, and requires C-terminal amidation for biologic activity.

Low levels of PYY are detected in enteroendocrine cells in the stomach, and levels increase distally along the small and large intestine, reaching their highest levels in cells in the colon and rectum (31). PYY is released from enteroendocrine L-cells lining the distal gastrointestinal tract in proportion to caloric intake. Plasma levels of PYY rise within 30 min of a meal and, in humans, circulating levels plateau at 1–2 h post-prandially, remaining elevated for up to 6 h (32). Protein-rich meals cause the greatest increase in PYY levels compared to other macronutrients (33, 34). The anorectic effects of PYY_3–36_ appear to be mediated centrally via the ARC. PYY_1–36_ and PYY_3–36_ exert their effects through the NPY family (35). PYY_1–36_ binds with similar affinity to NPY receptors. However, PYY_3–36_ is a selective high affinity at the Y_2_ receptor subtype (Y_2_R) (36) thought to be the receptor responsible for reduction of food intake by PYY. A vagal brainstem-mediated pathway may also be involved in the actions of circulating PYY_3–36_. PYY is present in enteric nervous plexus neurons innervating the gastrointestinal tract and the Y_2_R receptor has been identified on the vagus nerve (37), the effects of PYY_3–36_ on satiety and central control of appetite are clear. Most are mediated via anorectic neuronal populations in the ARC, but vagal/brainstem-mediated pathways and peripheral effects of PYY on gastric emptying and intestinal motility may also play a part.

#### Pancreatic polypeptide

Pancreatic polypeptide (PP) is an amidated 36-amino acid peptide that belongs to the “PP-fold” family of peptides. It is released post-prandially under vagal control by pancreatic islet PP cells (38). PP is comparable to other anorectic intestinal peptides such as PYY, being secreted in proportion to caloric intake.

Pancreatic polypeptides bind to all the members of the Y receptor family, but have the highest affinity for the Y_4_ receptor subtype (39). The effects of PP are likely to be mediated by the ventromedial hypothalamus (VMH) and paraventricular hypothalamus (PVN) and brainstem [area postrema (AP) and ARC] (40), in areas central to the control of appetite. The anorectic effects of PP in humans appear to be independent of changes in gastric motility (41).

#### Cholecystokinin

Cholecystokinin (CCK) is released post-prandially from the small intestine (42), and has also been shown to co-localize with PYY in L-cells (43). It is released in response to saturated fat, long chain fatty acids, amino acids, and small peptides that would normally result from protein digestion (44, 45). CCK1 receptors are present in peripheral tissues such as the pancreas, gallbladder, and on vagal afferent nerve fibers innervating the gut (46). Furthermore, CCK1 receptors have been identified in areas within the CNS involved in the regulation of food intake such as the NTS, AP, and dorsomedial hypothalamus (47). The CCK2 receptor has a different distribution. It is found in the cortex, hypothalamus, vagal afferents, and gastric mucosa, once again encompassing several areas known to be involved in appetite regulation. In humans, intravenous administration of physiological doses of CCK reduces food intake and increases the perception of fullness (48).

#### Leptin

Leptin is a 167-amino acid protein known to suppress appetite and regulate energy expenditure. Leptin is secreted mainly by adipocytes (49), but has also been found in the stomach (50) and the pituitary gland (51). Nevertheless, AT remains its main source responsible for 95% of leptin production (51). Circulating leptin levels are positively correlated with body mass index (BMI) and AT mass. Adipocytes possess large numbers of GH receptors (GHR) and it is known that GH directly regulates leptin gene expression (52). Furthermore, the production of leptin is influenced by several regulators, being stimulated by insulin and blood glucose but inhibited by sympathetic activity, lipolytic catecholamines, and free fatty acids (FFA). Leptin production correlates positively with AT mass (53) and is independent of the adiposity. In these reason, leptin levels are higher in women than in men (54). In humans, there is a highly organized pattern of leptin secretion over a 24-h period. In general, the circadian pattern is characterized by basal levels between 08:00 and 12:00 hours, rising progressively to peak between 24:00 and 04:00 hours, and receding steadily to a nadir by 12:00 hours (55). The nocturnal rise in leptin secretion is entrained to mealtime probably due to cumulative hyperinsulinemia of the entire day (54). Leptin is secreted in a regular pulsatile fashion with an interpeak interval of about 44 min, and the circadian rhythm is attributable solely to increased pulse height. Circulating leptin is transported across the BBB via a saturable process. Starvation reduces transport, whereas refeeding increases the transport of leptin across the BBB (56, 57).

Leptin is transported across the BBB by a saturable transporter system (58) and exerts its anorectic effect via the ARC, where both NPY/AgRP and POMC/CART neurons express leptin receptors (59). Leptin inhibits NPY/AgRP neurons and activates POMC/CART neurons (60, 61), resulting in reduced food intake. It also influences in the secretion of neuropeptides engaged in energy homeostasis such as CRH, TRH, and BDNF (62–65). Additionally, leptin stimulates the adrenergic system, thus increasing energy expenditure (66). In addition to its interaction with central neural processes, there is evidence of a synergy between leptin and the episodic satiety factor CCK discussed previously (67). Leptin has been shown to enhance the satiating effect of CCK (68). In addition, leptin-induced down-regulation of the hippocampal somatostatinergic system may potentiate its anorexigenic effect (69).

#### Amylin

Amylin is a 37-amino acid peptide, also known as islet amyloid polypeptide. In mammals, amylin is co-released with insulin from pancreatic β-cells in response to food intake and has an anorectic effect (70). Amylin seems to decrease food intake through both central and peripheral mechanisms and, indirectly, by slowing gastric emptying. The AP plays a predominant role in peripheral amylin’s satiating effect, involving a direct activation of AP neurons by blood-borne amylin. Its anorectic effect may, in part, be due to reduced expression of orexigenic neuropeptides in the LH area (71). There is evidence that amylin may also exert its effects through serotonergic, histaminergic, and dopaminergic systems.

#### Insulin

Insulin is produced in β-cells of the pancreatic islets of Langerhans and enters the brain from the circulation acting on hypothalamic neuron located mainly in the ARC, to reduce energy intake. There is also a specific insulin transport across the BBB regulated by a saturable mechanism that involves insulin receptors in brain microvessels. ICV infusion or systemic injection of insulin results in a dose-dependent suppression of food intake. Central action of insulin promotes anorexia because it decreases NPY and stimulates POMC expression (72). Insulin binds to its receptors highly expressed in POMC/CART and NPY/AgRP neurons. Insulin and leptin both activate POMC neurons, but they seem to differentially regulate AgRP, with leptin inhibiting and insulin stimulating its synthesis (73). Insulin deficiency is associated with increased NPY, while insulin administration inhibits hypothalamic NPY expression.

#### Glucagon-like peptides

Pre-proglucagon gene expression is limited to α-cells in the pancreas, L-cells in the gut, and neurons in the brain stem nucleus of the NTS. Whereas post-translational processing of proglucagon in the pancreas leads to the formation of glucagon and the major proglucagon fragment, proteolytic cleavage in the L-cells of the gut and in the NTS yields the peptides glicentin, oxyntomodulin (OXM), glucagon-like peptides 1 and 2 (GLP-1 and -2) (74). After a meal, GLP-1 and GLP-2 are secreted in parallel in the circulation. GLP-1, is perhaps best known as a gut-derived incretin hormone. GLP-1 is a 30-amino acid peptide and one of several cleavage products of the pre-proglucagon gene. It is secreted by the enteroendocrine L-cells of the distal intestine in response to incoming nutrients (75). GLP-1 is also a neurotransmitter synthesized by a small population of neurons in the NTS in the caudal brainstem (76). Food ingestion promotes the release of GLP-1 from L-cells in the intestine, which activates vagal afferents. (77). GLP-2 is a peptide highly conserved across different mammalian species. Its main biological effects are related to the regulation of energy absorption and maintenance of mucosal morphology, and function and integrity of the intestine. In the gastrointestinal tract, GLP-2 increases the uptake of luminal nutrients, including sugars and lipids, by augmenting the activity and expression of nutrients transporter. Its influence on appetite regulation is unclear but recent studies have shown that intraperitoneal injection of GLP-2 reduces food intake in mice (78).

#### Oxyntomodulin

Oxyntomodulin is a 37-amino acid peptide released post-prandially from L-cells in proportion to caloric intake. OXM (79) causes a reduction of neuronal activity in the ARC, PVN, and supraoptic nucleus. This pattern of activation is distinct from that of GLP-1 under the same conditions (80), implying that these two hormones act via different hypothalamic pathways. OXM reduces food intake in normal weight human volunteers when administered intravenously or subcutaneously (81). There is evidence that OXM may increase energy expenditure in humans (82).

#### Bombesin

Bombesin is a tetradecapeptide that was isolated from amphibian skin and is similar in structure to mammalian gastrin-releasing peptide (GRP) and neuromedin B (83, 84). Bombesin (85, 86) and GRP administration (87) decrease food intake in lean human subjects but not in obese women (88). Peripheral or central injection of bombesin reduces food intake that is not blocked by vagotomy (89, 90). Bombesin also activates the sympathetic nervous system (91). In animals that have been starved or have ventromedial hypothalamic lesions, bombesin produces a profound drop in temperature because the sympathetic nervous system cannot be activated (91, 92).

#### Obestatin

Recently, it has been demonstrated that preproghrelin undergoes additional proteolytic cleavage, generating a 23-amino acid peptide, which has been named obestatin. In contrast to ghrelin, obestatin has anorexigenic effects, reduces gastric emptying, inhibits jejunal contractions, and suppresses body weight gain (93). However, several recent studies performed in rats and mice under various experimental conditions did not reproduce these results (94, 95). Pan et al. (96) reported that obestatin is unable to cross the BBB and is rapidly degraded in the circulation; this was confirmed by Vergote et al. (97). An alternative hypothesis is that obestatin exerts its effects on eating and drinking through direct interactions with the gastrointestinal system. Indeed, Zhang et al. (98) observed decreased contractile activity of jejunum muscle strips *in vitro* and suppression of gastric emptying *in vivo* after obestatin treatment. Thus, the inhibition of jejunal contraction could generate an afferent vagal signal to induce satiety in the brain. Recently, Fujimiya et al. (99) supposed that obestatin may act on the obestatin receptor on vagal afferent nerve terminals, and corticotropin-releasing factor (CRF) and urocortin-2 neurons in the hypothalamus may mediate the action of obestatin to inhibit the gastroduodenal motility via CRF1-R and CRF2-R in the brain (100).

## Central Hypothalamic Peptides

The hypothalamus contains several important nuclei that are associated with energy homeostasis and feeding regulation. The LH is a feeding center, the VMH is the satiety center, and the ARC is an integrated center for feeding regulation.

### Hypothalamic orexigenic peptides

#### Neuropeptide Y

The ARC is the major site of expression for NPY within neurons in the hypothalamus that project to PVN, dorsomedial hypothalamus (DMH), LH, and other hypothalamic sites. Although NPY can produce diverse effects on behavior and other functions, its most noticeable effect is the stimulation of feeding after central administration (101). NPY synthesis in the ARC and its release into the PVN, the most abundant projection, is regulated by inhibitory afferent signals such as leptin and insulin and stimulatory signal as glucocorticoids. The NPY neurons are potential hypothalamic targets for leptin and inhibition of the synthesis, and probably release of NPY seems to partly explain the ability of leptin to induce hypophagia and weight loss. Insulin has been shown to inhibit NPY synthesis and secretion in the PVN. Five G-protein-coupled NPY receptors have been identified – Y_1_, Y_2_, Y_4_, Y_5_, and Y_6_. Y_5_ receptors have been implicated as important receptors that mediate the feeding effects of NPY (102, 103). The Y_5_ receptor is expressed at relatively high levels in the LHA, close to the site where NPY acts most potently to stimulate feeding (104).

#### Agouti-gene related protein

Agouti-gene related protein is a 132-amino acid peptide. Within the CNS, AgRP is expressed exclusively in the ARC and AgRP mRNA co-localizes with NPY mRNA in 95% of NPY positive cells in this nucleus (105). Rossi et al. have shown that like NPY, AgRP is an orexigenic peptide when injected ICV (106) or directly into the PVN or DMH (107). Uniquely, AgRP acts as an endogenous antagonist of the melacortin-3 (MC3R) and melacortin-4 receptor (MC4R) (108). It is likely AgRP plays a modulatory role in feeding. It may be that AgRP is more important during conditions of high energy requirements, such as pregnancy and lactation, under which it has been shown to be more highly expressed (109).

#### Melanin-concentrating hormone

Melanin-concentrating hormone is an orexygenic cyclic 19-amino acid neuropeptide. Within the hypothalamus (MCH), it is highly expressed in the LH and zona incerta (110) and has orexigenic effects after ICV infusion (111). Interest regarding the effector mechanisms by which MCH is orexigenic has largely focused on the MCHR1 receptors in the nucleus accumbens shell (AcbSh) where MCH injection decreases neuronal firing in medium spiny neurons (112). The nucleus accumbens is thought to be involved in motivational aspects of eating.

#### Hypocretins/orexins

The hypocretins (1 and 2; also known as orexins A and B) are excitatory neuropeptides that are produced in cell bodies of the LH area, but have extensive projections to many regions. The hypocretin/orexins bind to orexin receptor 1 and 2 (OXR1 and OXR2), which arise from two separate genes. The distribution of the two receptors is different. Within the hypothalamus, OX1R is the most highly expressed in the PVN (113).

Orexins are appetite-stimulating neuropeptides. Orexin neuronal cell bodies are present in the LH and DMH, and orexin-containing neuronal fibers are distributed in several nuclei, with abundant projections to the ARC. Orexin-containing neurons project to NPY-containing neurons in the ARC, and NPY neurons express the OX1R (114). Furthermore, orexins increase the cytosolic Ca^2+^ concentration in NPY neurons isolated from the ARC (115). These results indicate that NPY neurons receive excitatory signals from orexin-containing neurons in the LH. The distribution of the two receptors is also different; within the hypothalamus, OX1R is most highly expressed in the VMH and OX2R is most highly expressed in the PVN (113).

#### Galanin

Galanin is a 29-amino acid C-terminally amidated (30 amino acid, non-amidated in humans), found in the brain and the gut. Galanin coexists with GABA, noradrenaline, 5-hydroxytryptamine (5-HT), and NPY in several regions of the brain. Hypothalamic galanin neurology is found largely in the PVN, supraoptic nucleus of hypothalamus (SON), and ARC. Many galanin-positive fibers as well as galanin-positive neurons have been demonstrated in the dorsal vagal complex, suggesting that galanin produces its effects by involving vagal neurons. The nucleus of the solitary tract is the major source of the galanin terminals in the dorsal vagal complex. There are two cloned galanin subtype receptors: GalR1 and GalR2 are majorly distributed in the hypothalamus, PVN, amygdale, hippocampus, brainstem, spinal cord, peripheral nervous system, and other tissues (116). This peptide participates in modulating learning, memory, feeding, inflammation, pain threshold control, sexual behavior, insulin, and pituitary hormone release (117–119). Acute central administration of galanin has been reported to increase fat consumption.

#### Galanin-like peptide

Galanin-like peptide (GALP) is a novel 60-amino acid peptide, with residues 9–21 being identical to the biologically active N-terminal (1–13) portion of galanin (120). *In situ* hybridization studies have shown that GALP mRNA is distributed within the periventricular regions of the ARC (121, 122) in the median eminence, and in the pituitary gland of the rat (123). NPY-containing axon terminals are closely apposed (opposed) to GALP-containing neurons in the ARC (124). Moreover, Cunningham et al. (125) demonstrated, using double-label *in situ* hybridization, that GALP-containing neurons in the macaque expressed the NPY Y1 receptor, suggesting that NPY regulates GALP neurons in the ARC. However, whether NPY activates GALP is yet to be determined.

#### Cerebellin1

Cerebellin1 (Cbln1) is highly expressed in the hypothalamus. ICV administration of Cbln1 increases food intake and the release of NPY from hypothalamic explants and reduces plasma thyroid-stimulating hormone (TSH) levels after postinjection in rats without adverse behavioral effects. Cbln1 mRNA expression levels were increased in the ventromedial nucleus of the hypothalamus in fasted rats. These data suggest that Cbln1 is a novel orexigenic peptide, which may mediate its effects via hypothalamic NPY (126).

### Hypothalamic anorexigenic peptides

#### Cocaine and amphetamine-regulated transcript

Cocaine and amphetamine-regulated transcript is a neuropeptide which appears to be a powerful physiological anorexic signal. CART mRNA was identified on the basis of its increase following cocaine or amphetamine treatment in rats (127). CART peptide is localized in specific areas of the hypothalamus including the periventricular nucleus, dorsomedial nucleus, perifornical regions, lateral nucleus, and the ARC. In the PVN, CART mRNA is co-localized with vasopressin and CRF-containing neurons (128).

#### Melanocortins

The melanocortins are bioactive peptides derived from the precursor molecule POMC via tissue-specific post-translational cleavage (56). The POMC gene is expressed at physiologically significant levels in a number of mammalian tissues including anterior and intermediate pituitary, skin, the immune system, and hypothalamic neurons. The repertoire of products derived from POMC by any tissue is determined by the specificities of the convertases expressed in the tissue (129, 130). The intermediate lobe of the pituitary yields α-melanocytes-stimulating hormone (α-MSH), a peptide which activates melanocortin (MC) 3 and MC4 MC receptors and inhibits food intake. The MC3R and MC4R receptors are found in areas known to be involved in regulation of energy balance, but also in other regions such as cerebral cortex and hippocampus. Bioactive peptides generated in hypothalamic neurons act as endogenous ligands for the MC4R, a key molecule underlying appetite control and energy homeostasis (131).

#### Glucagon-like peptides

In the brain, release of GLP-1 within the nucleus of the solitary tract NTS and from projections of GLP-1 neurons to the PVN leads to GLP-1 receptor activation, which promotes satiety and anorexia. Activated GLP-1 neurons of the NTS also project to the ARC to modulate vagal motor outflow to the pancreas and other tissues not depicted, increasing insulin secretion from the β-cells in states of hyperglycemia and suppresses glucagon from the α-cells, leading to lowering of blood glucose. Systemic GLP-1 may also access the brain via leaks in the BBB such as the subfornical organ and the AP, as demonstrated to occur in rats. The intravenous administration of GLP-1 to normal and obese humans decreases food intake in a dose-dependent manner (132) as well as reducing gastric emptying (133, 134). These effects are thought to be mediated through vagal and brainstem pathways since peripheral administration of GLP-1 activates neurons within the brainstem in rats (1, 135).

The distribution of the co-localized peptide GLP-2 displays a perfect overlap with GLP-1 in the CNS, with the highest concentration in the diffuse ventral part of the dorsomedial nucleus (76, 136). When injected into the lateral ventricle, GLP-2 has a marked inhibitory effect on feeding. The effect of GLP-2 on feeding is both behaviorally and pharmacologically specific (76). The CNS GLP-2R is essential for the control of feed behavior. Glp-2r deletion in POMC neurons increases food intake with amplified meal frequency and accelerates gastric emptying, suggesting that CNS GLP-2 is a key satiety signal for the physiological short-term control of feeding behavior and gastric motility and contributes to the long-term homeostatic control of energy balance (or body weight). Moreover, activation of GLP-2R signaling suppresses food intake and gastric emptying through the MC4R signaling pathway. Guan et al. (137) findings suggest that gastric emptying is a key process for the short-term control of feeding behavior and POMC neuron-mediated suppression of food intake may be executed through decelerating gastric emptying (137).

#### Corticotropin-releasing factor

Corticotropin-releasing factor or CRH is a 41-amino acid mammalian neurohormone that is best known as the major physiological regulator of pituitary ACTH secretion. CRH is highly expressed in PVN neurons and, when centrally injected, inhibits food intake and reduces body weight in rats. Peripheral administration of human CRH increases energy expenditure and fat oxidation in humans. Leptin infusion stimulates CRH expression, while pretreatment with a CRH antagonist attenuates the leptin-induced reduction of food intake and body weight.

#### Neurotensin

Neurotensin (NT) is a 13-amino acid peptide. NT is produced in the ARC, PVN, and DMH of the hypothalamus and its microinjection into the PVN decreases food intake. NT neurons appear to play an anorectic role downstream of leptin as ICV leptin infusion into the PVN stimulates NT synthesis in association with reduced food intake (138, 139). These results suggest that leptin action may be mediated, at least in part, by NT.

#### Nesfatin-1

In the early 1990s, a protein was identified in mouse (140) and human cell lines (141) and termed nucleobindin or NEFA (DNA binding/EF-hand/acidic amino acid-rich region). Until now, two nucleobindins have been identified, namely nucleobindin1 (NUCB1) and nucleobindin2 (NUCB2 or NEFA). NUCB2 contains a 24-amino acid N-terminal signal peptide and a 396-amino acid sequence that is highly conserved in rodents and humans (142), pointing toward its physiological relevance. NUCB2 was localized on the plasma membrane and in the cytoplasm.

In 2006, Oh and colleagues (143) were the first to describe that putative post transcriptional processing of NUCB2 by the enzyme pro-hormone convertase (PC)-1/3 results in nesfatin-1 (amino acid 1–82), nesfatin-2 (amino acid 85–163), and nesfatin-3 (amino acid 166–396) (143). So far, the biological activity has only been demonstrated in nesfatin-1 and the fragment nesfatin-1_24–53_.

The initial report described the expression of NUCB2 mRNA substantiated by nesfatin-1 inmmunohistochemistry in rat hypothalamic and brainstem nuclei involved in the regulation of ingestive behavior, such as the PVN, supraoptic nucleus, ARC, LH, zona incerta, and NTS (143). Nesfatin-1 inmmunopositive neurons co-localize with a number of brain transmitters (8, 12–17).

Nesfatin-1 directly inhibits ARC neurons containing NPY. A recent study provided compelling evidence for the involvement of an oxytocin pathway in the inhibitory effect of nesfatin-1 on food intake (144). Nefastatin-1 is also likely to act in series through the recruitment of the central MC and corticotrophin-releasing factor 2 (CRF_2_) signaling systems to reduce food intake. The anorexic action of peripheral nesfatin-1/NUCB2 may be mediated by vagal afferents projecting to the nucleus of the solitary tract in addition to a potential hormonal action via crossing of the BBB (145).

Central nesfatin-1/NUCB2 mediates its anorexigenic effect via activation of melanocortin_3/4_ and CRF_2_ signaling and also by hyperpolarizing neurons containing the orexigenic peptide, NPY. Nesfatin-1 also activates the hypothalamic magnocellular oxytocinergic system, which could reduce food intake and delay gastric emptying. Peripheral nesfatin-1 can reach the brain via the circulation and crossing of the BBB and/or a direct action on circumventricular organs as well as on the modulation of vagal afferent activity.

Immunostaining in the peripheral tissues confirmed the expression of nesfatin-1/NUCB2 protein in the rat stomach and, additionally, in pancreatic endocrine islets of Langerhans, testis, and pituitary gland. Similarly, nesfatin-1-immunopositive cells of the endocrine pancreas exclusively co-localize with insulin in β-cells (146, 147). These findings suggest a differential release of nesfatin-1 and ghrelin from the stomach and nesfatin-1 and insulin from the pancreas, which warrants further investigation. The prominent and exclusive endocrine distribution of nesfatine-1/NUCB2 in cells of the stomach and pancreas support the fact that nesfatin-1 may act as a gut–brain peptide to influence food intake and glucose homeostasis. The anorexic action of peripheral nesfatin-1/NUCB2 may be mediated by vagal afferents projecting to the nucleus of the solitary tract, in addition to a potential hormonal action through crossing of the BBB (145).

## Pituitary Hormones

### Vasopressin

Vasopressin significantly reduces food intake over a 4 h period in experimental animals. The reduction in food intake, particularly in the first 30 min of feeding, is not significantly impaired by vagotomy, suggesting that its peripheral mechanism of action is different from that of CCK or enterostatin (148).

### Melanocyte-stimulating hormone

In yellow mice, that overexpress AgRP, treated with melanocyte-stimulating hormone (MSH) there is a substantial increase in food intake and weight gain which is 30–100 times greater than that of the acylated form (α) of MSH. In contrast, injection of αMSH produces a much more potent darkening of the melanocyte than does dMSH.

### Growth hormone

Following treatment with GH, hypophysectomized animals increase their food intake and growth. This finding could be a direct effect of GH on feeding centers or a may be due to a second stimulation by an enhanced flux of amino acids into new proteins that leads to an increase in feeding.

Growth hormone stimulates lipolysis in the AT and, particularly, in the visceral and sub-cutaneous depots (149–152). Hormone-sensitive lipase (HSL or LIPE) is a crucial enzyme implicated in this process. GH may also modulate the expression of the lipid droplet associating protein, CIDE-A (cell-death-inducing DFF45-like effector). CIDE proteins have been associated with lipid droplets, where they facilitate lipid accumulation and inhibit lipolysis. Unlike the AT, GH induces FFA uptake into skeletal muscle by up-regulation of LPL expression (153, 154). The re-sterification of triacylglycerides (TAG) from FFAs results in generation of intermediates such as diacylglycerol and ceramides that activate PKC isoforms. PKC can down-regulate insulin signaling by several mechanisms (155, 156). GH secretion is diminished in obesity, where increased FFA levels may have a suppressive effect on GH secretion. The hyperinsulinemia associated with this pathological situation may also contribute to decreased GH secretion (156). Thus, in this manner, the GH-induced increase in FFA uptake and TAG synthesis could result in insulin resistance. These data also suggest that GH induces a shift in substrate utilization from glucose to lipids in the skeletal muscle.

## Implications for Obesity and Metabolic Syndrome

### Obesity

The etiology of obesity is believed to be extremely complex and includes a combination of excess dietary calories and decreased physical activity, coupled with either some predisposing genetic factors or metabolic disorders (157–159).

#### Ghrelin

Although the role of ghrelin in the etiology of obesity is not understood, it is considered a vital target because of its capacity to induce a positive energy balance state (160, 161). Supporting the relevance of the ghrelin pathway regarding obesity, recent studies by Wortley et al. and Zigman et al. (162, 163) show that the absence of both ghrelin or its receptor GHS-1a protects mice against diet-induced obesity. In addition, ghrelin immunization in rats has been reported to reduce body weight gain (164) and catalytic anti-ghrelin antibodies in C57BL/6 mice reduce refeeding for 6 h after a 24-h starvation and maintain high levels of energy expenditure (165). Nevertheless, the immunization against ghrelin failed to cause long-term body weight reduction (166) and some studies with mice from a pure C57BL/6 background knockout for ghrelin or ghrelin receptor suggest only negligibly small differences in food intake and body weight under caloric restriction or a high-fat diet compared with wild-type mice (167). Finally, the absence of ghrelin in ob/ob mice does not seem to decrease food intake or body weight in this mouse model, although lowering blood glucose substantially (168, 169).

#### Leptin

Obese patients with three risk factors for metabolic syndrome have lower leptin levels (170). Mutations in the leptin receptor (*Ob-R*) gene are responsible for monozygotic obesity in rodents and humans. In obesity, there is also a defective transport of leptin across the BBB, which suggests the existence of central leptin resistance (171). This resistance could be inherited or secondary to obesity, associated with a less efficient transport of leptin via the BBB or abnormalities in leptin signaling (171).

#### Insulin

Insulin is a signal of satiety and obesity (172). Reduced expression or deletion of insulin receptors in the brain leads to hyperfagia and obesity (173).

#### Hypothalamic–pituitary–adrenal axis

There is a neuroendocrine integration of the stress centers in the CNS with centers that control appetite (174). Acute stress exerts an anorexigenic effect though stimulation of POMC/CART neurons by increased CRH levels and an additional decrease of NPY secretion (174, 175). CRH activates the hypothalamic–pituitary–adrenal axis with an increase in cortisol secretion which, in turn, inhibits the activation of the HPA axis. This is responsible for the anorexigenic effect of glucocorticoids in the case of acute stress. Chronic stress is associated with chronic activation of the HPA axis and prolonged glucocorticoid secretion. Chronically elevated levels of glucocorticoids exert orexigenic effects caused by inhibition of CRH and stimulation of NPY expression (174, 175). Several studies have also shown increased responsiveness of the HPA axis in obese patients to different stimuli and in a dynamic test with neuropeptides and small doses of dexamethasone (176). Abdominal obesity is also associated with attenuated negative feedback in the HPA axis (173).

#### Growth hormone

Growth hormone secretion is consistently reduced in obesity (177, 178). Consequently, low GH secretion could further contribute to accumulation of abdominal fat (156). Obesity-induced hyperinsulinemia, hypoadiponectinemia, leptin resistance, and increased bioactive insulin-like growth factor-1 (IGF-1) and FFA levels could suppress GH secretion from the pituitary by various mechanisms mentioned above. Reduced GH secretion further increases fat accumulation and, thus, exacerbates the obesity condition. Moreover, reduced GHR expression and increased expression of truncated GHR (ΔGHR) in the AT results in a GH-resistant state that also contributes to the complications associated with obesity (156).

#### Thyroid hormones

The hypothalamic–pituitary–thyroid (HPT) axis may play a direct role in appetite regulation. Hypothyroidism reduces energy expenditure and causes weight gain, while hyperthyroidism exerts the opposite effect (179). TRH, after peripheral or central administration, exerts a direct anorexigenic effect (180). Similarly, central administration of TSH causes inhibition of food intake in rats. Triiodothyronine (T3) which directly stimulates food intake at the hypothalamic level, can also cross the BBB and reach the CNS directly and then be transported to the ARC (181). Peripheral administration of T3 increases NPY and reduces POMC expression (179). Direct injection of T3 into the VMN also exerts an orexigenic effect in rats (179).

### Metabolic syndrome

Metabolic syndrome is due to cluster of cardiovascular risk factors that includes obesity, hypertension, insulin resistance, and glucose and lipid metabolic abnormalities (182, 183). Metabolic syndrome is associated with an increased risk of cardiovascular disease (CVD) and type 2 diabetes mellitus, even before the development of glucose intolerance (169, 183, 184).

#### Galanin

The effect of the galanin peptide family on the metabolic syndrome includes increased food consumption and the preference for a high-fat diet, which elevates the probability of obesity and dyslipidemia, and decreased insulin resistance and blood pressure to relieve the risk for type 2 diabetes mellitus and hypertension (185).

#### Adiponectin and leptin

Adiponectin, an anti-atherogenic and anti-inflammatory adipocytokine involved in glucose and lipid metabolism, improves insulin sensitivity (186). Lower levels of adiponectin were observed in patients with high blood pressure, hyperglycemia, low HDL-C, and hypertriglyceridemia, and in obese patients with MS (187). Brooks et al. showed that a low level of circulating adiponectin may be used as a possible biomarker for MS (188). Leptin, an anti-obesity adipocytokine, regulates body weight by modifying energy levels and increasing the metabolic rate while decreasing food intake. Most overweight and obese patients show resistance to leptin at the receptor level, and therefore, have higher leptin levels than non-overweight individuals (189). Serum leptin levels in patients with MS are higher than those in healthy controls (190). Adiponectin and leptin levels show an inverse correlation with each other (191).

García-Cardona et al. studied the correlation between obesity and insulin resistance and methylation frequency of the leptin and adiponectin promoters in obese adolescents, with the aim of identifying epigenetic markers that might be used as tools to predict and follow-up the physiological alterations associated with the development of the metabolic syndrome. Obese adolescents without insulin resistance showed higher and lower circulating levels of leptin and adiponectin, respectively, along with increased plasmatic concentrations of insulin and triglycerides. The methylation frequency of CpG sites located at −51 and −31 nt relative to the transcription start site of the leptin gene dropped dramatically in obese adolescents with insulin resistance (192).

#### Neuropeptide Y

Bray et al. studied positive association of an NPY gene variant (880I/D) with obesity in Mexican-American families (193). Additionally, there have been many studies examining the functional Leu7Pro polymorphism (rs16139). This single nucleotide polymorphism (SNP) has been associated with a large number of conditions related to obesity and metabolic syndrome traits, including increased BMI in adults (194), development of obesity in young adults (195), risk of hypertension (196), high plasma low-density lipoprotein-cholesterol (LDL-c) in children and adults (196, 197), and elevated plasma TAG (198). This variant has been associated with metabolic syndrome in patients with coronary artery disease (199). This SNP has also been shown to correlate with high birth body weight in preschoolers, the risk of an accelerated atherosclerotic process or carotid atherosclerosis in adults (196, 200), and the risk of type 2 diabetes mellitus in adults (201). Additionally, Josune-Olza et al. validated the association between the SNPs *NPY* rs16147 genotype and BMI in Spanish children, observing higher BMI values in TT homozygotes as compared with heterozygous C allele carriers (202).

#### Pro-opiomelanocortin

Yoo et al. (203) demonstrated that high POMC methylation in cord blood was associated with lower birth weight and children with high POMC methylation in cord blood showed higher TAG and higher insulin concentrations in blood. Thus, POMC methylation status in cord blood may be an early predictive marker of future metabolic syndrome.

## Conclusion

The control of energy balance depends critically on the CNS. The various CNS regions that control energy homeostasis are accessible to numerous circulating factors discussed above. Within these central locations are specific neuronal populations that recognize these signals and act in the network to integrate the multiple inputs, and help to regulate appetite (68). In particular, the hypothalamus is a center of integration of several nutrient signals. It can sense and integrate variations in adiposity and gastric hormones, as well as nutrients, and also receives neuroanatomical projections from other nutrient sensors, mainly within the brainstem. In addition, it also integrates these signals with cognitive forebrain-descending information to coordinate neuroendocrine, behavioral, and metabolic effectors of energy balance.

## Conflict of Interest Statement

The authors declare that the research was conducted in the absence of any commercial or financial relationships that could be construed as a potential conflict of interest.
